# Norway spruce somatic embryogenesis benefits from proliferation of embryogenic tissues on filter discs and cold storage of cotyledonary embryos

**DOI:** 10.3389/fpls.2022.1031686

**Published:** 2022-10-27

**Authors:** Sakari Välimäki, Caroline Teyssier, Mikko Tikkinen, Armelle Delile, Nathalie Boizot, Saila Varis, Marie-Anne Lelu-Walter, Tuija Aronen

**Affiliations:** ^1^ Production Systems, Natural Resources Institute Finland (Luke), Savonlinna, Finland; ^2^ Institut national de recherche pour Íagriculture, Íalimentation et Íenvironnement (INRAE), Office national des forê ts (ONF), BioForA, Orléans, France

**Keywords:** somatic embryogenesis (SE), *Picea abies*, forest regeneration material, desiccation tolerance, raffinose family oligosaccharides (RFO), cold storage, pre-maturation

## Abstract

Vegetative propagation opens opportunities for the multiplication of elite tree progeny for forest regeneration material. For conifers such as Norway spruce (*Picea abies*) the most efficient vegetative propagation method is seed multiplication through somatic embryogenesis. Efficient culture methods are needed for somatic embryogenesis to be commercially viable. Compared to culturing as clumps, filter disc cultures can improve the proliferation of embryogenic tissue (ET) due to more even spread and better developmental synchronization. In this study, ET proliferation on filter discs was compared to proliferation as clumps. The study comprised 28 genotypes in four trials. The benefits of adding a pre-maturation step and the selection of fresh ET for the subculture were evaluated. Pre-maturation on hormone-free media before maturation did not significantly improve embryo yield but improved greenhouse survival from 69% to 80%, although there was high variation between lines. Filter disc cultivation of ET did result in better growth than in clumps but was more dependent on ET selection and the amount of ET than the clump cultivation method. Filter proliferation also favors certain lines. Post-maturation storage can be used to change the storage compound composition of the produced mature embryos. The embryo storage compound profile was analyzed after post-maturation cold storage treatments of 0, 4, 8, 31, and 61 weeks and compared to that of the zygotic embryos. Cold storage made the storage compound profile of somatic embryos closer to that of zygotic embryos, especially regarding the raffinose family oligosaccharides and storage proteins. Sucrose, hexose, and starch content remained higher in somatic embryos even through cold storage. Prolonged storage appeared less beneficial for embryos, some of which then seemed to spontaneously enter the germination process.

## Introduction

The mounting pressure to eliminate fossil fuel consumption is increasing the need for efficient biomass production and thus the demand for high-quality forest regeneration material. Norway spruce [*Picea abies* (L.) Karst] is one of the most important timber species in Europe. However, its seed production is compromised by irregular flowering and pests (Nikkanen and Ruotsalainen, 2000; Pukkala et al., 2010). Vegetative propagation can be used to supplement the production of high-quality seed, and the most efficient vegetative propagation method for conifers is the multiplication of zygotic embryos by somatic embryogenesis (SE) (Bonga, 2015). SE enables the production of uniform and high-quality forest regeneration material (Denchev and Grossnickle, 2019), with potential to utilize genetic markers for the selection of desired traits (Park et al., 2016; Edesi et al., 2021).

Efficient and optimized culture methods alongside automation are needed to scale up the process, thus mitigating costs and making SE commercially relevant. Robust methods applicable to many cell lines are needed to maintain the genetic diversity of vegetatively produced forest regeneration material. For embryogenic tissue (ET) proliferation, multiple methods, including suspension cultures and bioreactors, may be used for scaling up (von Arnold et al., 2002; Egertsdotter et al., 2019; Välimäki et al., 2021), yet different semi-solid culture techniques need to be evaluated. The *in vitro* culture of ET on filter discs with *Pinus sylvestris* (L.) (Lelu-Walter et al., 2008; Aronen et al., 2009), *Pinus pinaster* (Ait.) (Lelu-Walter et al., 2006), and hybrid larches (*Larix* x *eurolepis* and *Larix* x *marschlinsii*) (Lelu-Walter and Pâques, 2009) leads to faster proliferation than cultivation as clumps on semi-solid media. This could be attributed to better culture dispersion and more even access to media (Mamun et al., 2018). However, a proliferation of ET on the filter can also affect both the number and quality of cotyledonary embryos produced later during the maturation phase (Aronen et al., 2009). For maturation itself, ETs are generally dispersed onto filter paper (Klimaszewska et al., 2001; Tikkinen et al., 2018a), which improves embryo yield compared with clump cultivation (Jiang et al., 2021). The transfer of ET from proliferation to maturation is often done through pre-maturation, which improves ET synchronization and response to maturation stimuli (Bozhkov et al., 2002). However, adding extra steps to the process increases costs, and the benefit of pre-maturation needs to be evaluated before implementation.

After maturation, embryos can immediately be germinated and transplanted. However, this may not be optimal for greenhouse survival (Tikkinen et al., 2018b). Desiccation treatments following maturation can be used to improve embryo quality (Högberg et al., 2001; Hazubska-Przybył et al., 2015), imitating drying that takes place in the late-stage development of zygotic embryos and priming the protein synthesis machinery for germination instead of embryo development (Attree et al., 1995). However, desiccation treatment adds another step to the process. A simpler alternative is to transfer the cotyledonary embryos on maturation plates into cold storage, which also improves the greenhouse survival of somatic embryos (Lipavská et al., 2000; Tikkinen et al., 2018b). In addition, the ability to store mature embryos makes large-scale production more flexible, because embryos can be produced throughout the year and then germinated before the growing season (Attree et al., 1995; Tikkinen et al., 2019). The carbohydrate changes that happen in embryos in cold storage and in soft desiccation treatments are very similar (Konrádová et al., 2003). These two treatments, applied at the end of maturation, induce stress, which triggers metabolic changes that lead to the acquisition of desiccation tolerance (Pond et al., 2002). These changes are decisive for the adaptation of the embryos to the subsequent steps for an orthodox seed: their desiccation and then their germination.

The aim of the present study was to investigate the possibilities of streamlining Norway spruce SE propagation. In four trials (Trials I–IV) using a total of 28 lines from 11 families, we studied the effect of ETs spread in a thin layer on filter discs and cultured as clumps on the proliferation rate and embryo production capacity (I and II). The benefits of a separate pre-maturation step (I) and the selection of only fresh ET for subculture and maturation (II) were evaluated. The application of filter disc and clump proliferation in a large-scale set-up to study their effect on production rate and the potential for commercial propagation were tested (III). The performance of the produced embryos was also followed through cold storage and germination up to their greenhouse performance (I, II). The consequences of cold storage duration (at +2°C for 4, 8, 26, and 61 weeks) for the storage compound quantity and profile of somatic embryos were analyzed (IV).

## Materials and methods

### Plant material

The Norway spruce embryogenic cell lines (later referred to as lines) were initiated in 2014 according to Klimaszewska et al. (2001) from immature seed embryos extracted from cones from full-sib families of progeny-tested plus trees. The lines were cryopreserved, thawed, and maintained as described by Varis et al. (2017). The lines used in each trial are presented in **Table 1**. In Trial IV, pooled samples of zygotic embryos extracted from cold-stored seeds ready for planting (Seed Orchard 170) were used for comparison.

**Table 1:** SE lines and families used in different trials.

	SE lines			
Family	Trial I	Trial II	Trial III	Trial IV
E462 x E64	3492			3416
E9 x E3231		4262		4334
E799 x E1366	5137	5111, 5129	5111, 5113, 5115, 5129, 5137	5111
E207 x E252		1375		
E162 x E81		290		
E2105c x E2283	1548	1548		
E1551 x E2229		4631		
E18 x E436		623, 655	602, 605, 607, 629, 649, 650, 655, 660	
E207 x E1373	1130	1130		
E46 x E3222	2816	2816, 2851	2803, 2816, 2851, 2872, 2891, 2894	
E9 x E1361				4024

### Proliferation

The ET was grown on semi-solid Litvay’s modified medium (mLM) (Litvay et al., 1985; Klimaszewska et al., 2001) according to Varis (2018) with 1% (w/v) sucrose and half concentrations of macro-elements with pH adjusted to 5.8. The plant growth regulator 2,4-dichlorophenoxyacetic acid (2,4-D) and 6-benzylaminopurine concentrations were 10 and 5 µM respectively. The media was solidified with 4 g/l gellan gum (Phytagel, Sigma Aldrich), and 500 mg/l L-glutamine was added after autoclaving by filter sterilization. The media (21 ml per plate) was dosed in 92 x 16 mm sterile Petri dishes (Sarstedt). Until it was used in the trials and as control treatments (referred to as selected clumps), ET subculturing was done every 2 weeks by selecting newly grown fresh ET and placing it onto new media as clumps.

**Figure 1:**
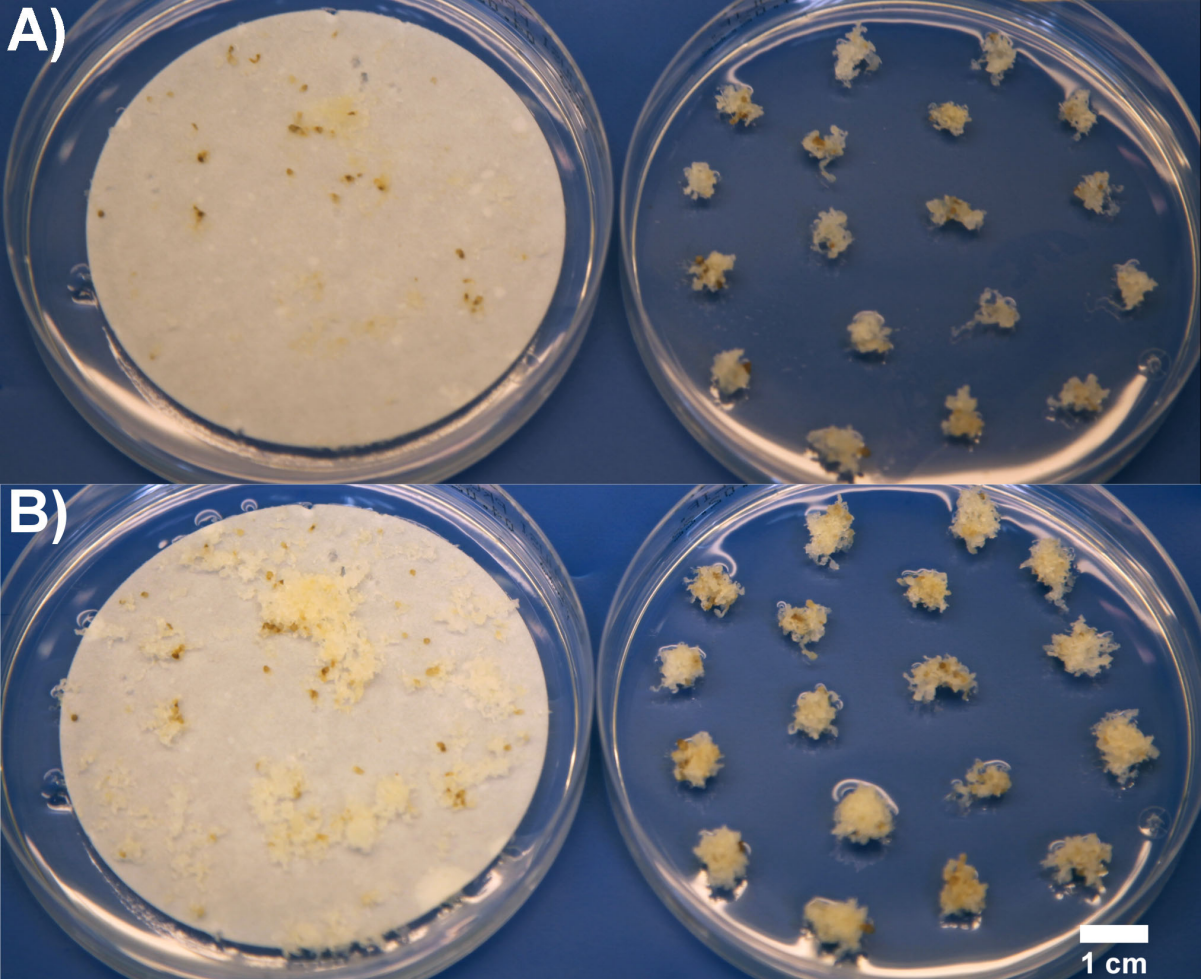
ET of line 2891 immediately after subculture **(A)**, and one-week after subculture before transfer to maturation **(B)** on filters (left) and as clumps (right) in Trial III.

The proliferation of the ET as clumps and spread on filter discs (Munktell no. 1, diameter 7 cm, Ahlstrom-Munksjö, Falun, Sweden) placed on semi-solid media was compared in Trials I, II, and III (**Table 2**; **Figure 1**). In Trial I, all subculturing was done by selecting fresh ET for fresh media, either on filter discs or as clumps. The filter disc cultures in Trial I, were done based on the protocol by Lelu-Walter et al. (2006) by suspending around 200 mg ET into a sterile polypropene tube (13 ml, VWR) filled with 5 ml of hormone-free liquid culture medium. The tube was shaken vigorously, and the contents poured onto a filter disc placed in a Büchner funnel and dried with a low-pressure pulse. The filter paper with ET was then placed onto fresh proliferation media. The fresh ET was meticulously picked from the clumps using forceps. Clump cultures were proliferated in plates filled with ca. 20 clumps weighing a total of around 700 mg. Every line in every treatment was proliferated on three replicate plates.

**Table 2:** Proliferation and maturation results from Trials I and II.

	Proliferation treatment	Inoculum size (mg)	Growth rate	ET weight	Maturation treatment	Embryos/gFW
**Trial I** (5 lines) Three proliferation plates and nine maturation plates were done for each line in each treatment.	unselected clumps	690–770	3.44 ± 0.07 (a)^1^	2.58 ± 0.05	–	58.3 ± 6.74 (a)^2^
					pre-mat.	77.1 ± 13.7 (ab)^2^
	selected filters	190–211	7.83 ± 0.37 (b)^1^	1.56 ± 0.07	–	92.8 ± 10.0 (ab)^2^
					pre-mat.	99.0 ± 13.2 (b)^2^
**Trial II** (12 lines) Three proliferation plates and nine maturation plates were done for each line in each treatment.	selected clumps	301–348	4.35 ± 0.10 (ab)^3^	1.37 ± 0.04	selected ET	65.5 ± 7.32 (a)^4^
	unselected clumps	303–353	4.28 ± 0.12 (a)^3^	1.37 ± 0.04	unselected ET	55.2 ± 6.83 (a)^4^
					selected ET	78.9 ± 9.94 (ab)^4^
	selected filters	299–347	4.77 ± 0.14 (b)^3^	1.51 ± 0.05	selected ET	98.9 ± 8.98 (b)^4^
	unselected filters	301–358	3.97 ± 0.16 (a)^3^	1.29 ± 0.05	unselected ET	60.2 ± 7.52 (a)^4^

^1^Mann-Whitney U-test, normal distribution not assumed.

^2^Welch ANOVA with Games-Howell as post hoc, data transformation to square root for normal distribution.

^3^Welch ANOVA with Games-Howell as post hoc (data normally distributed, equal variances not assumed).

^4^One-Way ANOVA with Bonferroni as post hoc. The growth rate was calculated as fresh weight (FW) at 14 days/FW of inoculum. Embryogenic tissue (ET) weight stands for the FW of all ET on the plate after 14 days of culture. Embryos/gFW stands for counted cotyledonary somatic embryos adjusted for 1 g (FW) of ET placed for maturation. The mean values are presented with ± standard error of the mean. Different letters (a, b) indicate significant differences between the treatments. The results for each line in each treatment are presented in **Supplementary Tables 1–4**.

In Trial II, two rounds of selected subculturing were done for both clumps and filter discs as in Trial I, except the amount of subcultured ET was around 300 mg for all treatments (**Table 2**). Selective subculturing was compared with subculturing without selection. The unselected clumps were subcultured by dividing them into smaller pieces, which were placed on fresh media. A stencil with 13 marked spots was used to keep subculturing uniform throughout the trial. The unselected filter disc cultures were first started by picking full clumps instead of selected ET for dispersion. For the subculture, ET was picked from a random sector of the filter disc using a stencil. If insufficient ET was present in the given sector, picking continued clockwise to the next sector.

In the more practice-oriented Trial III, the focus was on evaluating the suitability of filter culture for large-scale production. When cryopreserved and thawed ET started to grow, it was divided into clump and filter cultivation. The subculturing of the filter cultures was carried out by picking most of the tissue from the plates, suspending it in hormone-free liquid mLM in a 50 ml sterile tube, and pipetting 5 ml aliquots of suspension onto filter papers, which were dried in a Büchner funnel with a low-pressure vacuum pulse and transferred to fresh proliferation media. Clumps were subcultured by picking all the fresh ET for fresh proliferation plates. The cultures were compared based on how soon it was possible to conduct 40 maturations from each line and based on maturation yield as embryos/g of fresh weight (FW).

### Maturation

In all trials, maturation was done according to Tikkinen et al. (2018a) by suspending fresh ET from proliferation media into 3 ml hormone-free liquid maturation medium and pouring the suspension onto a filter paper (Munktell no. 1, diameter 5.5 cm) in a Büchner funnel. The filter paper was dried with a low-pressure pulse and placed on a semi-solid maturation medium with 60 g/l sucrose and 30 µM (±)-abscisic acid (ABA).

In Trial I, a one-week pre-maturation treatment on hormone-free mLM maturation medium was tested for both selected clump and filter disc cultures. Pre-maturation (1 week, +25°C, in darkness) was done by transferring the clumps or filter discs with ET onto pre-maturation media. ET from the same proliferation plates was used one-week earlier for maturations without pre-maturation. Fresh ET was selected from the pre-maturation for the maturations, which were carried out as previously described.

In Trial II, besides using the fresh ET from selected clumps and selected filters, ET from unselected clumps was randomly picked for maturation by picking whole clumps, and from unselected filters by picking ET from a random sector of the filter disc. In addition, ET from unselected clumps was picked selectively for maturation. The maturation time was 7 to 8 weeks, and all good morphology (at least four cotyledons, straight and intact stem) cotyledonary somatic embryos were counted from the plates, after which the plates were moved to cold storage (darkness, +2°C). The embryos from Trial I were stored for 22 to 23 weeks, from Trial II for 3 to 4 weeks, and from Trial IV for 4, 8, 26, and 61 weeks. In Trials I and II, the number of cotyledonary embryos was counted again after cold storage (6 months in cold storage in Trial I and less than a month in cold storage for Trial II).

In Trial III, 40 maturations were made from both clumps and filters (selecting fresh ET) in five two-week intervals. The results were analyzed based on how soon after thawing sufficient ET for 40 maturations was available. In Trial III, cotyledonary embryos were counted from a randomly selected sample of the plates to estimate the potential difference in embryo production between filter and clump proliferation. The samples were three random plates from the first set of five maturations for each line in each treatment, and then one random plate for every five maturations.

### Germination and transplantation

All the good morphology cotyledonary embryos from Trials I and II were germinated for 1 to 2 weeks in 190–210 μmol/(m^2^ s) photosynthetic photon flux under Valoya L35 spectrum AP673L Clear LED lights (Valoya Oy, Helsinki, Finland), with an 18 h/6 h day/night photoperiod (Tikkinen et al., 2018b). The embryos were transplanted into Plantek 81F containers (81 separate ventilated compartments of 85 cm^3^) filled with peat-based substrate in a greenhouse in July 2021. Survival (alive or dead) was evaluated from photographs taken 41 days after transplantation.

### Storage compound analysis

The embryos were matured for Trial IV as previously described from the selected clumps. Embryos matured for 7 weeks (five samples of 50 mg for the protein sample and three samples of 70 mg for the carbohydrate sample) were snap-frozen in liquid nitrogen at four different timepoints: immediately after the maturation period, 4, 8, 31, and 61 weeks into cold storage. The samples were stored at −80°C until analysis. Zygotic embryos and megagametophyte were extracted from *Picea abies* mature seeds. Soluble proteins were extracted from five biological replicates of samples as described by Teyssier et al. (2014). About 50 mg FW of frozen material was extracted with 0.5 ml of lysis buffer containing 2% (w/v) polyvinylpolypyrrolidone (PVPP, Sigma), 5% (v/v) β-mercapto-ethanol, 2% v/v sodium dodecyl sulfate (SDS), 50 mM Tris HCl (pH 6.8), and 10% (v/v) glycerol. The samples were extracted twice. Protein content was determined using the Bradford assay, with bovine serum albumin as a standard. The results (mean ± standard error of five biological repetitions) were expressed as soluble protein content in µg/mg FW. The same quantity of extracted proteins (15 µg) was separated by SDS-polyacrylamide gel electrophoresis (SDS-PAGE) on a 12% gel with stacking gel (4%), following standard protocols. The gel was stained for proteins with colloidal Coomassie Brilliant Blue G-250 (CBB-G). Soluble carbohydrates and starch were identified and quantified following Gautier et al. (2018). Briefly, ethanolic supernatants of powdered extract from lyophilized samples (initially 70 mg FW) were purified using activated charcoal (Merck) and PVPP, dried, suspended in water, and injected into a Chromaster high-performance liquid chromatography system (VWR Hitachi) equipped with a Rezex™ RPM-Monosaccharide Pb^+2^ (8%) column (Phenomenex), then eluted with ultrapure water at a flow rate of 0.6 ml/min. The carbohydrates in the eluates were quantitatively detected with an evaporative light scattering detector (ELSD) 85 (VWR Hitachi), and the peak areas were electronically integrated using OpenLAB CDS EZChrom (Agilent). The carbohydrates were identified by co-elution with standards (Sigma), quantified from calibration curves and expressed in mg/g dry weight (DW). The samples’ starch content was determined in glucose equivalents by analyzing amyloglucosidase hydrolysates of residual pellets of the extracts after soluble carbohydrate extraction (Gautier et al., 2019) using the “*D-glucose assay procedure”* kit (Megazyme). Each sample was assayed in triplicate.

### Statistical analysis

ET growth and embryo yield were tested for normality with the Kolmogorov-Smirnov and Shapiro-Wilk tests, and for homogeneity of variance with the Levene test. If the data were not normally distributed, a non-parametric test (Mann-Whitney U-test) was used. Data with unequal variances were analyzed with Welch analysis of variance (ANOVA), with Games-Howell as *post hoc*, and transformed to square root for normality if required. Normally distributed data with equal variances were analyzed with one-way ANOVA with Bonferroni *post hoc*. The statistical tests used for each dataset in Trials I and II are specified in **Table 2**, and when filter and clump cultivation were compared in Trial III, the Mann-Whitney U-test was used. Greenhouse survival in Trials I and II was analyzed with logistic regression, and treatment, line and placement in growth containers (row, column) were used as covariates. The location of each plant inside the container was not significant and was therefore omitted from the final models. The biochemical analyses in Trial IV were analyzed with one-way ANOVA and with multiple comparisons of means with Tukey contrasts. The percentage of effect factor was calculated as a percentage of the sum squares. Hierarchical ascendant cluster analysis (HCA) was calculated using Ward’s minimum variance clustering method, and the Euclidian distance as a measure of similarity. In all cases, the differences were considered significant when *p* < 0.05. The statistical analysis was carried out using IBM statistics 27 or R software (version 4.1.3, ^©^ 2009–2022 RStudio, PBC).

## Results

### Proliferation

The highest ET growth rate in relation to inoculum size was achieved in Trial I with filter disc cultures and ca. 200 mg inoculum (**Table 2**; **Supplementary Table S1**). In Trial II, the filter culture inoculum was increased to around 300–350 mg, but ET obtained after 2 weeks did not increase as in Trial I. In Trial I, a large (690–770 mg) inoculum was used for ET cultured as clumps, resulting in limited growth in relation to inoculum mass (**Table 2**; **Supplementary Table S2**). In Trial II, more comparable inoculum was used for both clump and filter disc cultures than in Trial I, and no significant differences were found in growth rates.

**Figure 2:**
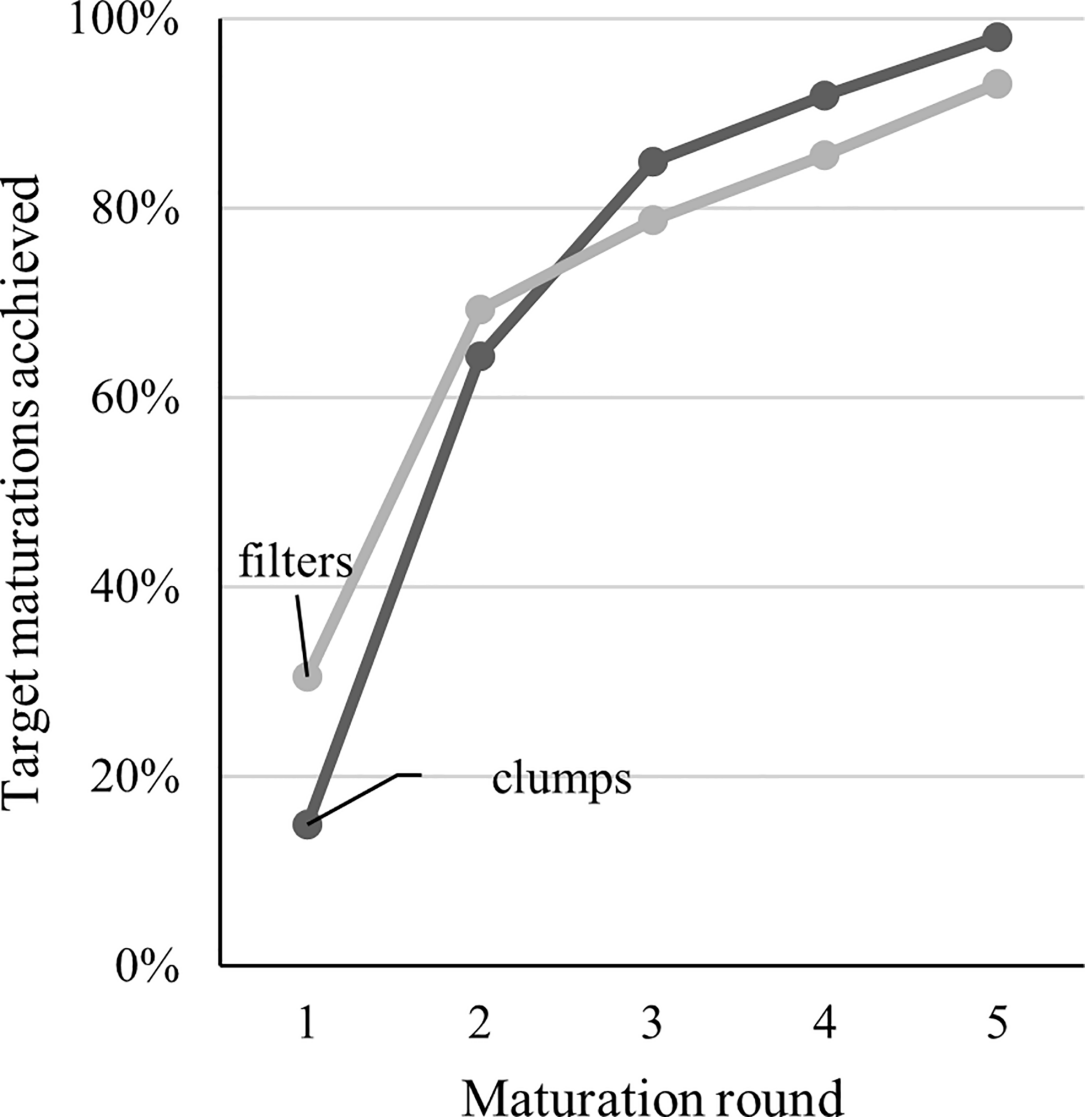
Visualization of maturation progress in Trial III with two weeks between each maturation round. In Trial III, the goal (100%) was to get 40 maturation plates from all 19 lines used in the trial for both clump and filter proliferation. The factor limiting the progress was the amount of ET in the proliferation plates.

The selection of fresh ET for the subculture significantly improved proliferation in the filter but not in the clump cultures (Trial 2, **Table 2**). At the beginning of Trial III, the filter disc cultures initially produced more ET, and a larger number of maturations was gained, but the difference was levelled after the second round of maturations (**Figure 2**). Eventually, a slightly higher proportion of the goal (40 maturations per line) was achieved with clump-cultured ET.

### Maturation yield

In Trial I, embryo yield was significantly higher from pre-matured ET grown on filters than from ET grown as clumps and matured without pre-maturation (**Table 2**; **Supplementary Table S1**). Without pre-maturation, filter-cultured ET produced more embryos, but the difference was not significant. Embryo yield from selectively subcultured and matured ET grown on a filter disc was superior to the other treatments in Trial II (**Table 2**; **Supplementary Table S4**). However, the difference was not significant compared to ET subcultured without selection as clumps but selected for maturation.

More cotyledonary embryos were counted after cold storage in Trial I (+37% after six months of cold storage) and II (+18% after 28 days of cold storage) than at the end of the maturation period. The exact embryo numbers from each line and treatment are presented in **Supplementary Tables S5**, **S6**.

**Figure 3:**
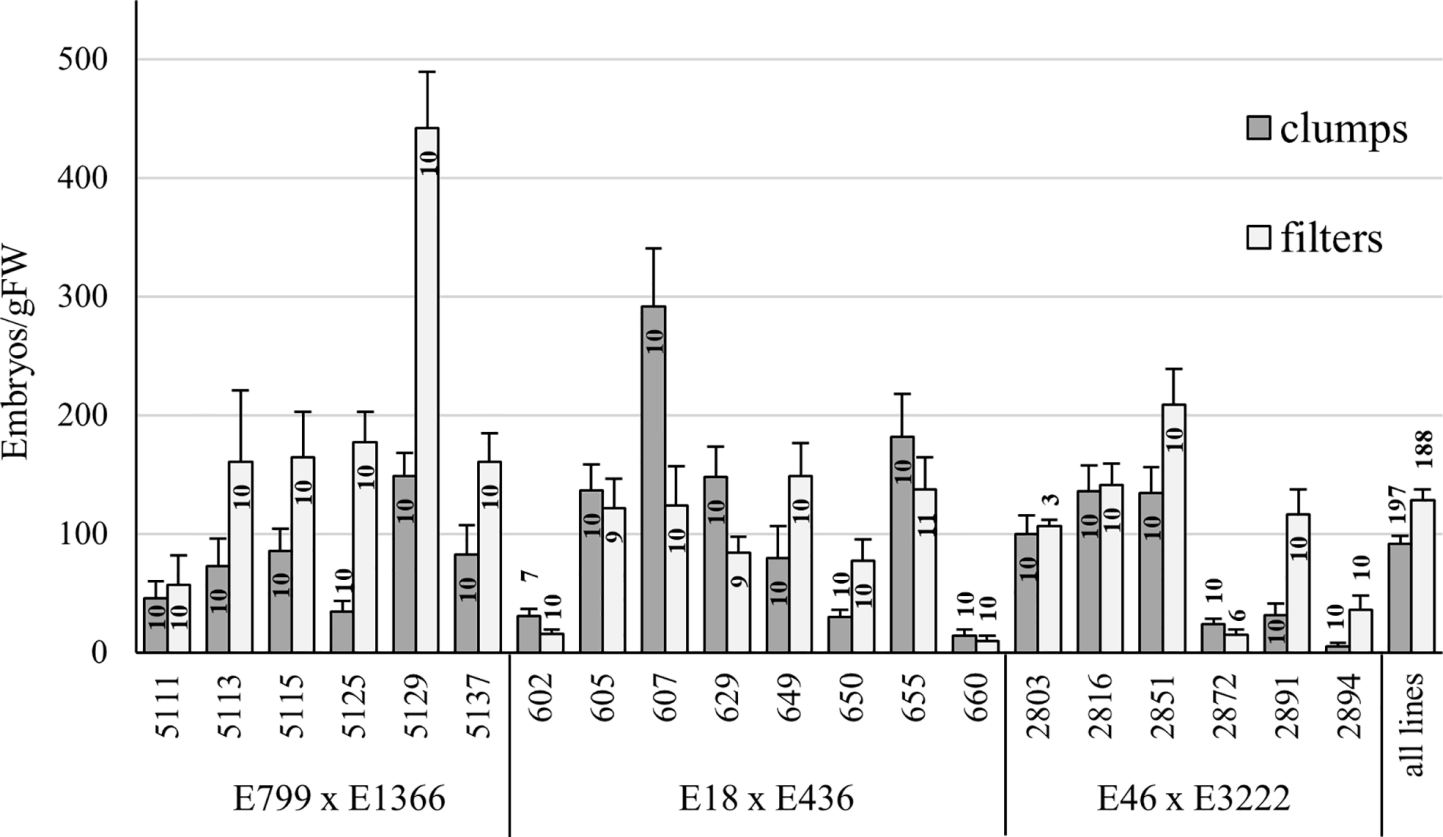
Embryo yield from Trial III, comparing ET proliferated on filter discs or as clumps. Mean embryo yield and standard error of the mean for a line and overall are presented. The number of plates (n) for each line in each treatment is presented inside each bar.

In Trial III, filter disc cultures produced significantly more embryos than clump-grown ET (*p* < 0.01, Mann-Whitney U-test) (**Figure 3**). However, some lines performed significantly better when ET was matured from clump cultures. Lines from three families were used in Trial III, and lines from two families produced significantly more embryos after proliferation in filter (*p* > 0.01 for both) than in clumps. From one family, embryo yield was higher from clump-grown ET, but the difference was not significant (*p* = 0.47).

### Greenhouse survival

**Figure 4:**
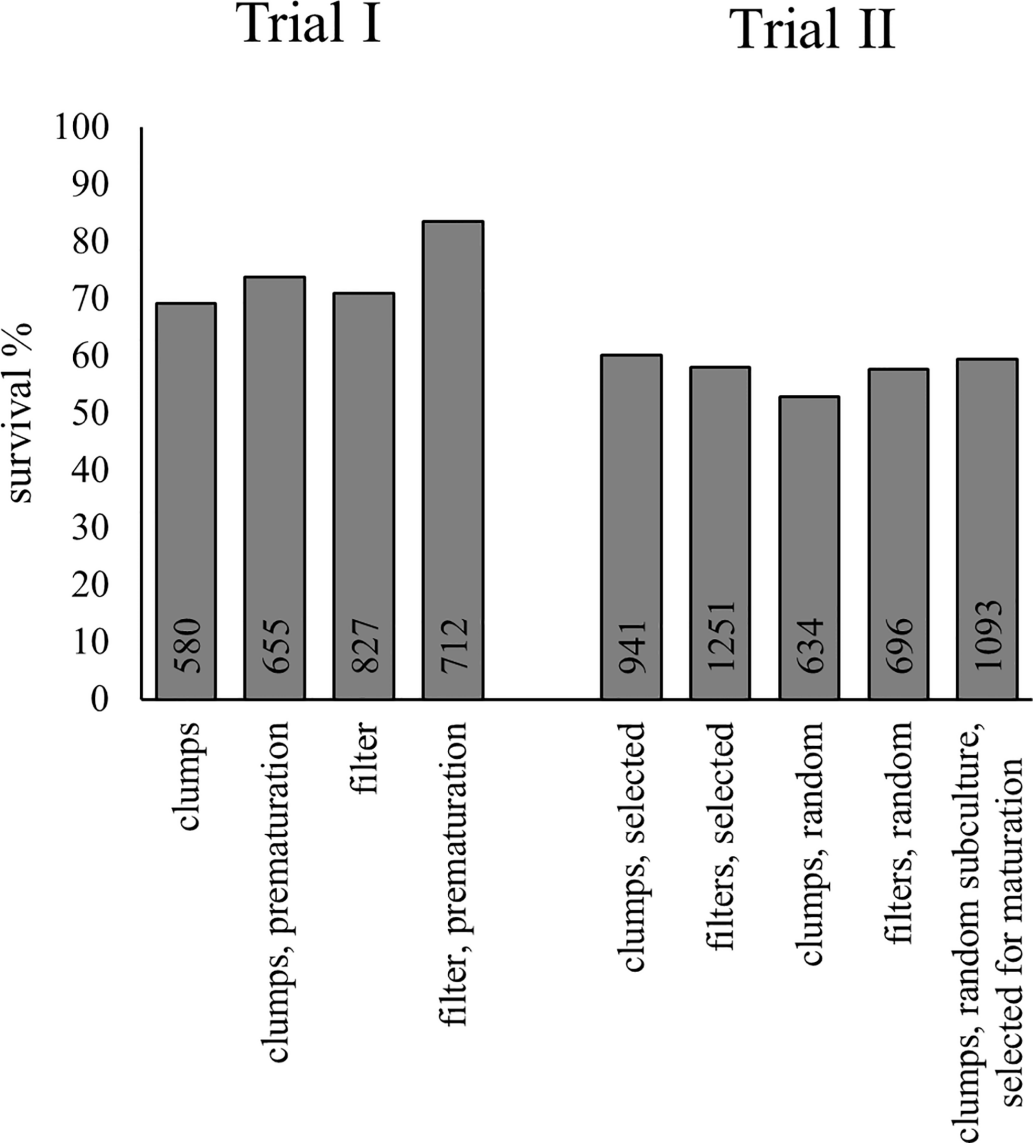
Greenhouse survival of embryos from Trials I and II. The survival was evaluated from photographs 41 days after transplantation. The number of embryos transplanted from each treatment is presented inside the base of the columns.

In Trial I, pre-maturation increased the greenhouse survival of embryos, especially from ET cultivated using filter discs (**Figure 4**; **Supplementary Table S7**). The number of cases predicted correctly by the model was improved only when pre-maturation status was applied as a covariate with line (**Table 3**). With pre-maturation, embryo survival was 80%; without pre-maturation, it was 69% when data from filter- and clump-originated embryos were combined. The effect of culturing method (clumps or filter discs) was not significant and did not improve the predicted cases and was excluded from the final model. The overall survival in Trial I was 75%.

**Table 3:** Logistic regression model of greenhouse survival after 41 days in Trial I.

Model, log(p/1-p)	Variable	P-value	Odds ratio (95% CI)	Treatment or line	% of cases predicted correctly by model
	*log(p/1-p) =*1.042 *+* 0.623p - 0.738l_1_ + 0.9661_2_ - 1.637l_3_ – 0.354l_4_				78.6
	Prematuration or not	< 0.001	1.864 (1.498–2.320)		
	Line	< 0.001	Reference	1130	
		< 0.001	0.478 (0.329–0.696)	1548	
		< 0.001	2.626 (1.637–4.213)	2816	
		< 0.001	0.195 (0.138–0.275)	3492	
		0.073	0.702 (0.476–1.034)	5137	

Proliferation method (selected clumps or filters), pre-maturation status (yes or no), line, and placement in growth containers (row, column) were used as covariates. The proliferation method and the location of the individual plant inside the container were not significant and were omitted from the final model.

In Trial II, overall survival was 58%, which is lower than in the previous trial, probably because of extensive liverwort growth in multiple containers. The difference between treatments was small (**Figure 4**; **Supplementary Table S8**), but there were many differences between lines. Line was the only variable, which improved correctly predicted cases, indicating the minimal effect of either proliferation treatment or SE selection on embling survival (**Table 4**). The effect of location in the container (row or column) was excluded from both final models, as it was not significant and did not improve the models’ prediction.

**Table 4:** Logistic regression model of greenhouse survival after 41 days in Trial I.

Model, log (p/1-p)	Variable	P-value	Odds ratio (95% CI)	Treatment or line	% of cases predicted correctly by model
*log(p/1-p) =*1.319 *+* 0.593t_1_ + 0.145t_2_ + 0.843t_3_ - 0.752l_1_ + 0.0.9441_2_ - 1.613l_3_ – 0.371l_4_					71.2
	Treatment	0.041	Reference	Proliferation as clumps, fresh ET selected	
		0.112	0.853 (0.701–1.038)	Proliferation as filters, fresh ET selected	
		0.272	0.878 (0.697–1.107)	Proliferation as clumps, ET taken randomly	
		0.006	0.732 (0.585–0.915)	Proliferation on filter discs, ET taken randomly	
		0.837	0.979 (0.798–1.201)	Proliferation as clumps, ET taken randomly for proliferation, selected for maturation	
	Line	< 0.001	Reference	290	
		< 0.001		623	
		< 0.001		655	
		< 0.001		1130	
		< 0.001		1375	
		< 0.001		1548	
		< 0.001		2816	
		< 0.001		2851	
		< 0.001		4262	
		< 0.001		4631	
		0.187		5111	
		< 0.001		5129	
					

Treatment, line, and placement in growth containers (row, column) were used as covariates. The location of the individual plant inside the container was not significant and was therefore omitted from the final model.

### Storage compound analysis

**Figure 5:**
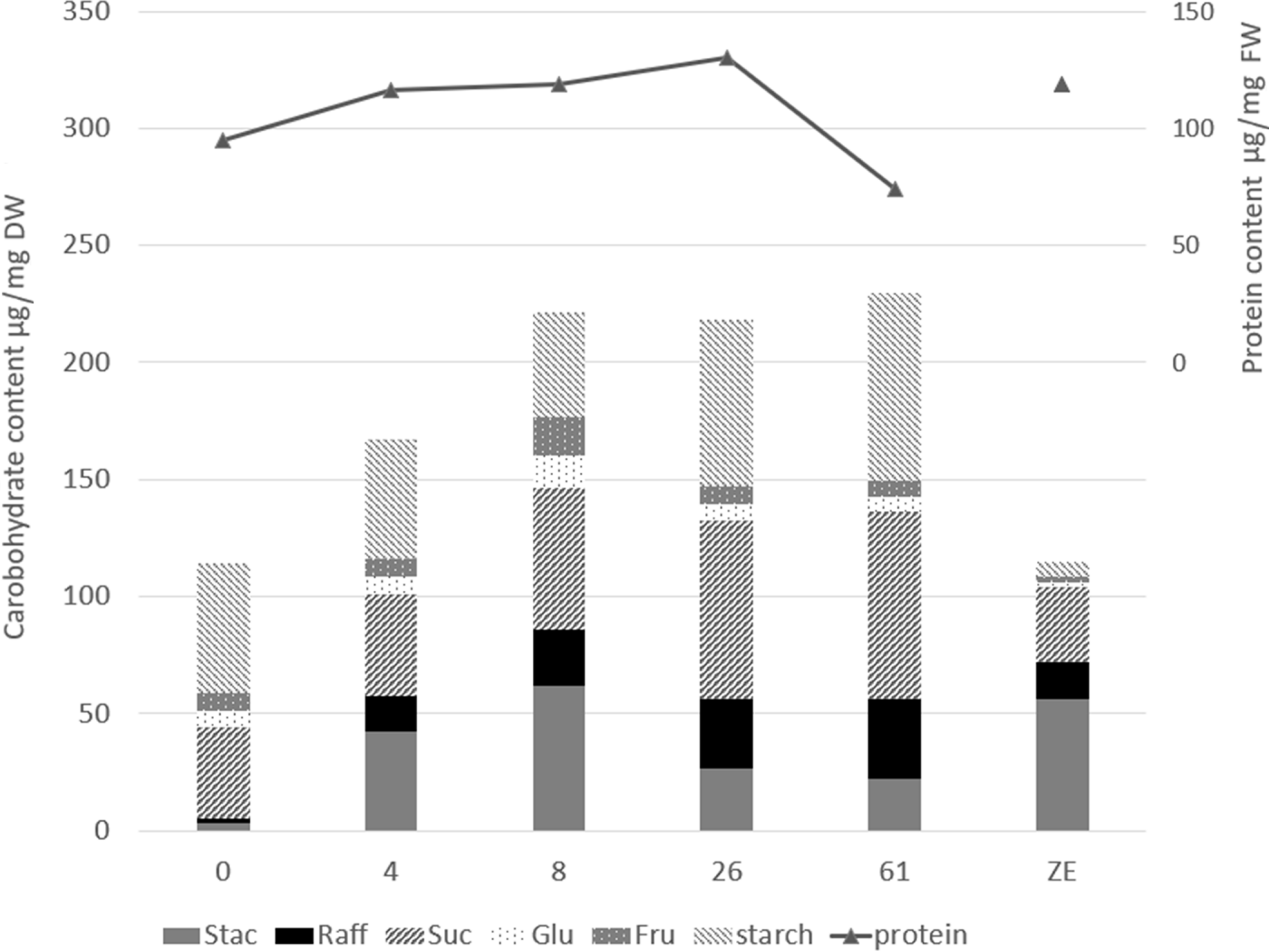
Storage compound content of the cotyledonary somatic embryos at the end of maturation (0), after 4, 8, 26, or 61 weeks of cold storage (4, 8, 26, or 61), or in the mature zygotic embryos (ZE). Values are means of five replicates for carbohydrate assay (on the left axis) and three replicates for protein assay (on the right axis). The statistical results of the multiple comparisons of means are given in the (**Supplementary Table 10**). Stac, stachyose; Raff, raffinose; Suc, sucrose; Glu, glucose; Fru, fructose; DW, dry weight; FW, fresh weight.

In Trial IV, the storage compounds considered were total proteins, soluble carbohydrates, and starch. Their quantities fluctuated considerably in somatic embryos, depending mainly on the duration of cold storage and to a lesser extent the genotype (*p* < 0.01, storage effect of 80.0% or 74.3% and genotype effect of 5.4% or 5.7% for proteins and carbohydrates respectively (**Supplementary Table S9**; **Figure 5**).

**Figure 6:**
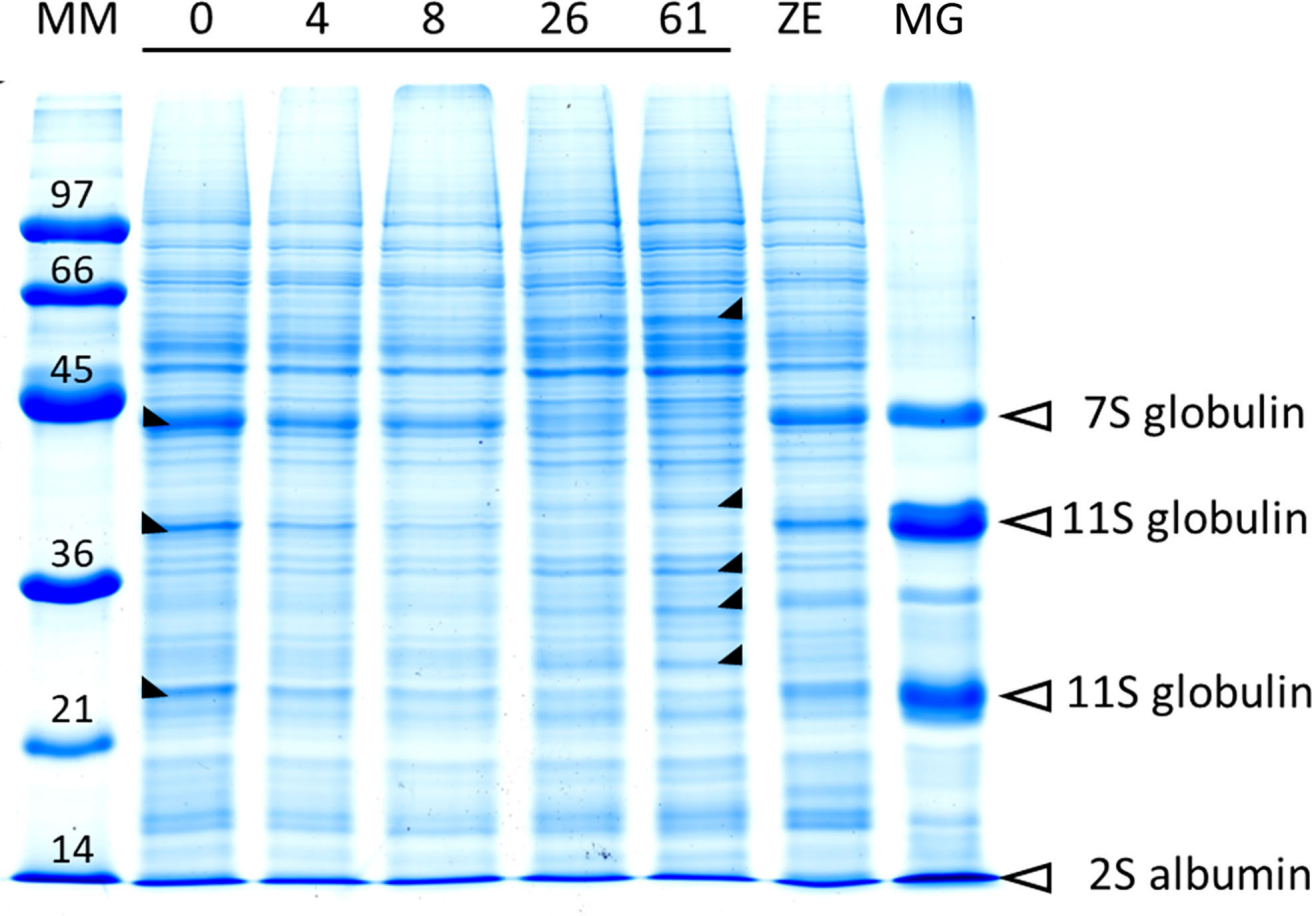
SDS-PAGE total protein profile in somatic embryos (line 3,416) according to cold storage time (from 0 to 61 weeks), compared with the zygotic embryo (ZE) profile. Molecular weights of protein markers (kDa) are given in the left-most line (MM). The location of major storage proteins in the protein pattern, given by the megagametophyte (MG) profile, is indicated with arrows. The major modifications of the protein abundances induced by the storage time are indicated on the corresponding bands by a black triangle (ψ increase; ξ decrease).

**Figure 7:**
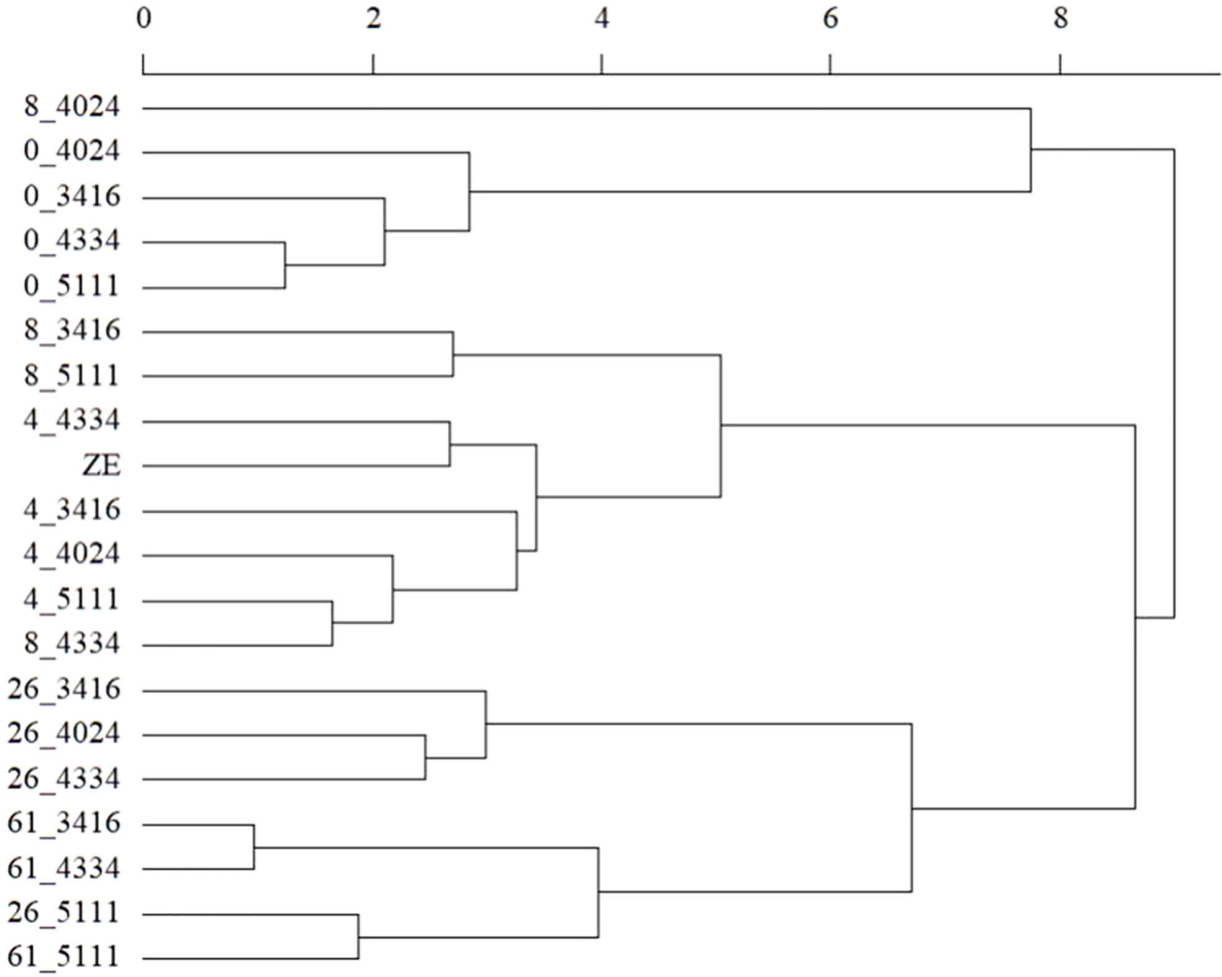
Hierarchical clustering analysis of zygotic embryos (ZE) and stored cotyledonary somatic embryos sampled at the end of maturation or after 4, 8, 26, or 61 weeks of cold storage (0, 4, 8, 26, and 61 respectively) of the cell lines 3416, 4024, 4334 or 5111. Clustering was based on the entire available quantitative dataset (fructose, galactose, glucose, maltose, sucrose, myo-inositol, chiro-inositol, stachyose, raffinose, and starch and total protein content).

The protein content in cold-stored embryos increased during storage, reaching the level of zygotic embryos at 4 weeks (**Figure 5**; **Supplementary Table S10**). However, somatic embryo protein content dropped significantly between 26 and 61 weeks of storage, and at 61 w, it was lower than before cold storage. The embryo protein content also changed qualitatively, as demonstrated by the SDS-PAGE gel in **Figure 6**. The change was related to the storage time, and mainly concerned the storage proteins which disappeared after 8 weeks of storage. From 26 weeks, all globulins were depleted, whereas other proteins were synthetized. Simple carbohydrate content (fructose, galactose, glucose, chiro-inositol, and myo-inositol) did not change with storage time, in contrast with sucrose content, which increased with storage time (**Figure 5**; **Supplementary Table S10**). The raffinose family oligosaccharides (RFOs) were practically absent at the end of maturation. Cold storage rapidly increased RFO content to the level of RFOs in zygotic embryos. Raffinose accumulated throughout the storage; stachyose did so only during the first weeks of storage and then decreased between 26 and 61 weeks. Starch content initially decreased (4 or 8 weeks) and then increased compared to mature embryos before cold storage. Somatic embryos always had a higher starch content and lower stachyose content (except for 8 weeks) than zygotic embryos. According to the carbohydrate profile of the samples, cold storage for 4 weeks seemed to be the treatment that approximated somatic embryos most to zygotic embryos. This was confirmed by the HCA, (**Figure 7**) calculated on all 11 quantitative components (individual sugars and proteins). The principal clusters separated the samples mainly according to the duration of the treatment, separating the short from the long.

## Discussion

The highest ET growth rate was achieved with filter disc cultivation and 200 mg inoculum (Trial I). However, the total ET mass it was possible to maintain on a single proliferation plate was lower for filter disc cultures than for cultures grown as clumps. In Trial II, using inoculum of 300–400 mg in both filter disc and clump cultures resulted in a similar growth rate for both proliferation methods. Thus, faster bulk up of ET can be achieved with filter disc cultures, but more ET can be maintained as clumps. No benefit is achieved for filter disc cultures from an increase of inoculum from 200 to 300 mg. On the contrary, even higher growth rates may be achieved with lower inoculum, which is the case for *Pinus sylvestris* and *P. pinea* (Aronen et al., 2009; Carneros et al., 2009). In Trial III, a more production-oriented approach was taken to evaluating filter disc cultivation. Filter disc subcultures were conducted based on visual estimation without weighing the ET. Despite this, filter disc cultures initially produced more ET, and thus material for almost double the maturations on the first maturation round compared with clump proliferation. though filter disc cultures sustained lower ET mass, more of it was fresh growth suitable for maturation. In addition, on filter discs ET is loose on the paper and easy to collect, whereas pulling fresh ET from clumps takes more time and attention. By the end of the Trial III, a slightly higher proportion of target maturations was achieved from clump cultures. The higher amount of ET in clump cultures may allow them to regenerate more between the maturation rounds and be more viable in the prolonged upkeep and multiple rounds of subculture. Moreover, when using filter discs for either ET proliferation or maturation, the quality of the paper can affect the results depending on the preferences of the propagated material (Pullman et al., 2003).

In all trials, filter-proliferated ET produced more embryos than ET proliferated as clumps, but the difference was not always significant. The embryo yield from different treatments varied among lines, with some lines producing more embryos from clumps and some from filters. In Trial III, which had the most lines, a significant difference between filter- and clump-grown ET was found. Lines from three crossings were used in the trial, and the lines from family E799 x E1366 especially benefited from filter cultivation. Differences in embryo yield between and within lines are commonly observed when testing SE, which highlights the need for trials with enough lines.

The selection of fresh ET from the clumps’ periphery did not significantly improve proliferation. Meticulous selection of ET for subculture is recommended for better ET quality (Denchev and Grossnickle, 2019). However, only two rounds of subcultures were conducted and the difference might have become visible with a longer culture period. On the other hand, with filter cultures, selecting fresh ET improved the proliferation rate. In Trial III, the selection of ET was done, but more superficially, which may explain the decline in filter disc cultures after the first round of subcultures. The selection of fresh ET in proliferation and maturation had a greater impact when matured from filter culture than from clumps. This indicates that at least some degree of selection of ET is necessary for successful maturation, especially when plating ET for maturation.

Pre-maturation increased cotyledonary embryo yield and nursery survival, especially when embryos were matured from filter grown ET. Proembryogenic tissues predisposed to proliferation inducing plant growth regulators, especially auxin 2,4-D, are less responsive to maturation (Garcia et al., 2019). Pre-maturation on hormone-free medium is often recommended to mitigate transfer from proliferation to maturation (von Arnold et al., 2002). Activated charcoal or alternative carbon sources can also be used to enhance pre-maturation (Denchev and Grossnickle, 2019). Pre-maturation can synchronize ET development and prime ET for maturation triggered by ABA and increased osmotic stress on maturation media stimuli (Filonova et al., 2000; Bozhkov et al., 2002; Tikkinen et al., 2018a; Garcia et al., 2019). In *Picea abies*, suspension cultures rinsing the ET placed on the filter in a Büchner funnel before suction and plating for maturation is instrumental for maturation success (Välimäki et al., 2021). Based on the results, pre-maturation may improve greenhouse survival for otherwise poorly performing lines, such as 3492 in Trial I. Although this is an additional step in the process, it may be justified to capture a larger number of lines and ensure genetic diversity in production, without increasing plant loss during growing in nursery. In this study, pre-maturation was conducted for whole cultures after the removal of ET for maturation. It is possible that some cultures did not completely regenerate during pre-maturation, and that less ET was available for maturation from the pre-maturation plates than from the initial proliferation plates.

More embryos were available for germination after cold storage than before it. This is a novel finding, which indicates post-maturation development of embryos in cold storage conditions. As the cultures are not synchronized, some embryos are not properly developed after 7–8 weeks of maturation, but continue their development in cold storage. Some cotyledonary embryos also go through precocious germination during prolonged cold storage, but cold storage still appears to have a net benefit for embryo yield.

Cold can induce the acquisition of desiccation tolerance (Pond et al., 2002; Konrádová et al., 2003; Clark et al., 2010; Liao and Juan, 2015), which is beneficial to germination (Maruyama and Hosoi, 2012; Liao and Juan, 2015). The acquisition of this tolerance is obtained through radical modifications of the cells’ biological processes, taking them from maturation to preparation for germination. This may explain the better greenhouse survival of cold-stored embryos (Tikkinen et al., 2018b). Acquisition of desiccation tolerance is characterized at the molecular level mainly by an increase in storage reserves, the synthesis of molecules protecting the molecular structures from desiccation, the synthesis of proteins involved in the germination and a drastic decline in ABA, a germination inhibitor (Hoekstra et al., 2001). According to Liao and Juan (2015), cold treatment applied to *P. abies* cotyledonary embryos under conditions similar to ours leads to a drastic decline in ABA from 6 weeks of storage.

During cold storage, the carbohydrate and protein content of the somatic embryos increased. The carbohydrate spectrum shifted with an increase in sucrose, which is favorable to germination and the regulation of osmotic pressure (Attree et al., 1991; Bomal et al., 2002; Businge et al., 2013). The observed accumulation of stachyose and raffinose plays a vital role in the protection against damage during desiccation (Lipavská et al., 2000). The increase in RFOs after a short cold storage period is in line with Konrádová et al. (2003). As RFOs were practically absent in somatic embryos at the end of maturation, cold storage brought their storage compound profile closer to that of zygotic embryos. However, the carbohydrate storage (measured in per mg of DW) of somatic embryo doubled the proportion of carbohydrate reserves in somatic compared to zygotic embryos. The starch and sucrose content remained high and even slightly increased with all somatic lines during cold storage. The higher starch content in somatic than in zygotic embryos is in line with results obtained with *P. glauca* (Joy et al., 1991). In general, the sucrose and starch content in somatic embryos seems to be positively correlated with the sucrose and ABA content of the culture medium (Businge et al., 2013; Hazubska-Przybył et al., 2016), and their content strongly decreases when mature somatic embryos of *P. abies* are maintained in the absence of culture medium (Eliášová et al., 2022). In our study, since there was no input of ABA or sucrose during storage, the observed increases in sucrose and starch must be related to one or more other cold-induced biological processes, which may be the origin of the degradation of some stachyose into raffinose from 26 weeks of storage.

Cold storage induced a slight increase in protein content up to 26 weeks of storage. This slight increase confirms that the accumulation of protein reserves in conifers occurs mainly during maturation (Joy et al., 1991; Cui et al., 2021; Eliášová et al., 2022), even before the acquisition of desiccation tolerance. In our study, the cold-induced protein content modification was also qualitative, with a significant reduction in certain storage proteins and the appearance of a few bands. While the embryos were stored on their nutrient-rich maturation medium, the decrease in storage proteins did not reveal a consumption of storage reserves for the functioning of the cells, but it marked the initiation of germination for the embryos (Misra et al., 1993; Bornman et al., 2003; Businge et al., 2013), which was more evident from 26 weeks. The appearance of new proteins, or the increase of already present proteins, in relation to the duration of storage may correspond to the synthesis, or consolidation, of proteins specific to protection against desiccation damage (late embryogenesis-abundant proteins, heat shock proteins, proteins related to oxidative stress, etc.) or to germination (establishment of photosynthesis, root, and hypocotyl elongation).

Thus, cold storage would contribute to the observed better greenhouse survival of somatic embryos in Tikkinen et al. (2018b) through all the induced molecular modifications, bringing them, regardless of the storage time, closer to zygotic embryos. This proximity, highlighted by the hierarchical clustering analysis, is more pronounced with the embryos stored for 4 or 8 weeks. Storage for up to 8 weeks therefore seems suitable for *Picea abies* somatic embryos, which is the case for *P. glauca* embryos (Pond et al., 2002), to induce the acquisition of desiccation tolerance and its favorable effects on germination. The more pronounced disappearance of the storage proteins and the reduction in stachyose from 26 weeks of storage prompt us to limit the storage of the embryos to a shorter period. Indeed, cold storage did not lead to water loss (results not shown). Moreover, the non-quiescent embryos continued to evolve, as shown by the profiles of the storage compounds. This phenomenon has already been observed for embryos kept in maturation for too long, which then seemed to enter germination (Lelu-Walter et al., 2008). This leads to the mobilization of storage proteins (a decline in protein content at 61 weeks) and lipids, in particular triglycerides, the hydrolysis of which leads to the formation of starch (Jordy and Favre, 2003). The unexpected start of germination when the culture conditions are not optimal leads to a decrease in germination rates when the germination step is triggered (Bornman et al., 2003; Tikkinen et al., 2018b).

In conclusion, filter disc culture can improve the ET growth and cotyledonary embryo yield of *P. abies.* However, proliferation as clumps appears to be more consistent in capturing as many lines as possible and more suitable for prolonged culture upkeep. The benefits of filter disc cultures in increased growth and embryo yield could be most efficiently achieved when it is used for bulking up the tissue immediately before maturation, and at least superficial selection of ET should be implemented, both for subculture and maturation. The pre-maturation step could be considered to improve the greenhouse survival of poorly performing lines. Cold storage makes the storage compound profile of somatic embryos closer to that of zygotic embryos. The increase in RFOs during cold storage can trigger the process of acquisition of the desiccation tolerance, which redirects the embryo cellular machinery from maturation to germination, thus improving greenhouse survival. For optimal results, this storage should be short (up to 8 weeks) to ensure the germination itself is not initiated if the environmental conditions are not optimal. However, longer storage is not detrimental and can be done if necessary to spread the workload throughout the year for larger production volume.

## Data availability statement

The raw data supporting the conclusions of this article will be made available by the authors, without undue reservation.

## Author contributions

SakV had the main responsibility for designing and conducting the filter disc experiments and analyzing their results. MT, SaiV and TA participated in designing the experiments and data analysis. CT, MT, and TA planned, and CT, AD, and NB carried out, the storage compound analysis. SakV and CT did the interpretation of the storage compound data. SakV and CT wrote the first draft of the manuscript, and MT, SaiV, M-AL-W, and TA participated in writing the manuscript. All the authors have read and agreed to the published version of the manuscript.

## Funding

This research was funded by the European Regional Development Fund, South Savo Regional Council, and Savonlinna municipality (A76396). The MULTIFOREVER project supported this research under the umbrella of the ERA-NET co-fund Forest Value by ANR (FR), FNR (DE), MINCyT (AR), MINECO-AEI (ES), MMM (FI), and VINNOVA (SE). Forest value has received funding from the European Union’s Horizon 2020 research and innovation program, under agreement N°C 773324.

## Acknowledgments

We would like to thank the staff at Luke’s Savonlinna unit who were involved in lab and greenhouse work for their excellent technical assistance.

## Conflict of interest

The authors declare that the research was conducted in the absence of any commercial or financial relationships that could be construed as a potential conflict of interest.

## Publisher’s note

All claims expressed in this article are solely those of the authors and do not necessarily represent those of their affiliated organizations, or those of the publisher, the editors and the reviewers. Any product that may be evaluated in this article, or claim that may be made by its manufacturer, is not guaranteed or endorsed by the publisher.

## Abbreviations

2,4-D, 2,4-dichlorophenoxyacetic acid; ABA, abscisic acid; ANOVA, analysis of variance; DW, dry weight; ET, embryogenic tissue; FW, fresh weight; HCA, hierarchical ascendant cluster analysis; mLM, Litvay’s modified medium; PVPP, polyvinylpolypyrrolidone; RFO, raffinose family oligosaccharide; SDS, sodium dodecyl sulfate; SDS-PAGE, sodium dodecyl sulfate polyacrylamide gel electrophoresis; SE, somatic embryogenesis.
