# Supplementary material for: Norway spruce somatic embryogenesis benefits from proliferation of embryogenic tissues on filter discs and cold storage of cotyledonary embryos
Source: Front Plant Sci. 2022 Oct 27;13:1031686. doi: 10.3389/fpls.2022.1031686 (PMC9647157; doi:10.3389/fpls.2022.1031686)
Supplement: Supplementary file 1 [file DataSheet_1.docx]

# Supplementary Data

Supplementary Table 1. Line specific proliferation rates in Trial I. Proliferation rate was measured as inoculum (FW) / FW after 2 weeks from subculture. The results are given as means ± SEM. The number of replicate plates for each line in each treatment is 6.

| Line | Selected Clumps | Selected Filters |
| --- | --- | --- |
| 1130 | 3.22 ± 0.16 | 8.02 ± 0.25 |
| 1548 | 3.77 ± 0.07 | 9.4 ± 0.29 |
| 2816 | 3.15 ± 0.11 | 7.25 ± 0.45 |
| 3492 | 3.26 ± 0.17 | 5.09 ± 0.93 |
| 5137 | 3.81 ± 0.09 | 9.39 ± 0.31 |
| Total | 3.44 ± 0.07 | 7.83 ± 0.37 |

Supplementary Table 2. Line specific embryo yield as embryos / g of ET (FW) in Trial I. The results are means from six maturation plates ± SEM, except for lines 4262 (random filters 9 plates) and 623 (selected clumps 5 plates)

| Line | Selected Clumps | Selected Filters | Selected Clumps, prem. | Selected Filters, prem. |
| --- | --- | --- | --- | --- |
| 1130 | 63.75 ± 12.19 | 62.39 ± 15.14 | 19.48 ± 6.26 | 41.16 ± 9.72 |
| 1548 | 90.77 ± 17.7 | 117.15 ± 11.09 | 35.34 ± 10.84 | 75.71 ± 9.31 |
| 2816 | 34.05 ± 17.8 | 89.63 ± 7.74 | 61.94 ± 29.77 | 161.6 ± 41.49 |
| 3492 | 65.82 ± 12.97 | 175.85 ± 20.3 | 184.76 ± 42.32 | 162.09 ± 56.79 |
| 5137 | 37.11 ± 9.42 | 18.77 ± 12.43 | 83.88 ± 13.32 | 110.37 ± 27.23 |
| Total | 58.3 ± 10.04 | 92.76 ± 6.74 | 77.08 ± 13.66 | 99.00 ± 13.21 |

Supplementary Table 3. Line specific proliferation rates in Trial II. The results are given as means ± SEM.

| Line | Selected Clumps | Nonselected Clumps | Selected Filters | Nonselected Filters |
| --- | --- | --- | --- | --- |
| 290 | 3.98 ± 0.02 | 3.71 ± 0.20 | 3.10 ± 0.15 | 2.07 ± 0.17 |
| 623 | 4.49 ± 0.10 | 4.18 ± 0.16 | 5.00 ± 0.81 | 3.74 ± 0.15 |
| 655 | 4.26 ± 0.18 | 4.28 ± 0.07 | 5.44 ± 0.29 | 4.45 ± 0.25 |
| 1130 | 4.03 ± 0.06 | 3.94 ± 0.19 | 5.37 ± 0.40 | 5.52 ± 0.12 |
| 1375 | 2.88 ± 0.11 | 2.73 ± 0.03 | 2.56 ± 0.15 | 1.63 ± 0.16 |
| 1548 | 5.58 ± 0.09 | 5.78 ± 0.22 | 6.50 ± 0.14 | 6.38 ± 0.30 |
| 2816 | 4.30 ± 0.15 | 3.97 ± 0.03 | 4.85 ± 0.23 | 3.76 ± 0.42 |
| 2851 | 5.34 ± 0.22 | 5.33 ± 0.23 | 5.55 ± 0.26 | 5.33 ± 0.23 |
| 4262 | 5.08 ± 0.15 | 4.85 ± 0.21 | 5.65 ± 0.41 | 4.50 ± 0.08 |
| 4631 | 3.42 ± 0.17 | 3.17 ± 0.15 | 4.59 ± 0.03 | 4.05 ± 0.18 |
| 5111 | 4.24 ± 0.28 | 4.93 ± 0.30 | 4.49 ± 0.28 | 2.57 ± 0.45 |
| 5129 | 4.23 ± 0.05 | 4.24 ± 0.21 | 4.24 ± 0.65 | 2.50 ± 0.28 |
| Total | 4.36 ± 0.12 | 4.30 ± 0.14 | 4.78 ± 0.20 | 3.88 ± 0.25 |

Supplementary Table 4. Line specific embryo yield as embryos / g of ET (FW) in Trial II. The results are means from six maturation plates ± SEM, except for lines 4262 (random filters 4 plates) and 623 (selected clumps 5 plates)

| Line | Selected Clumps | Nonselected Clumps | Nonselected clumps in prolif., ET selected for maturation | Selected Filters | Nonselected Filters |
| --- | --- | --- | --- | --- | --- |
| 290 | 8.82 ± 4.54 | 5.65 ± 1.39 | 4.09 ± 1.85 | 20.49 ± 4.77 | 16.93 ± 6.27 |
| 623 | 106.73 ± 40.81 | 83.08 ± 9.21 | 108.49 ± 23.30 | 79.49 ± 16.08 | 59.85 ± 21.28 |
| 655 | 123.58 ± 18.40 | 74.55 ± 34.17 | 137.2 ± 44.02 | 201.84 ± 13.88 | 100.9 ± 20.85 |
| 1130 | 43.31 ± 16.20 | 98.05 ± 13.74 | 211.22 ± 18.50 | 64.5 ± 21.44 | 80.93 ± 14.46 |
| 1375 | 142.92 ± 31.80 | 96.93 ± 31.58 | 104.67 ± 21.56 | 191.04 ± 22.44 | 93.42 ± 25.06 |
| 1548 | 3.44 ± 5.30 | 19.57 ± 1.54 | 16.01 ± 5.43 | 71.41 ± 7.33 | 24.85 ± 18.24 |
| 2816 | 19.36 ± 11.92 | 40.35 ± 10.27 | 32.56 ± 7.42 | 91.66 ± 4.86 | 9.14 ± 24.95 |
| 2851 | 52.11 ± 32.73 | 84.48 ± 11.03 | 85.81 ± 59.16 | 103.51 ± 36.02 | 177.74 ± 16.41 |
| 4262 | 47.66 ± 9.04 | 27.24 ± 9.10 | 78.63 ± 32.82 | 50.61 ± 5.61 | 9.22 ± 20.97 |
| 4631 | 48.21 ± 6.39 | 28.55 ± 10.99 | 29.92 ± 2.78 | 35.98 ± 10.69 | 27.16 ± 6.67 |
| 5111 | 55.72 ± 9.83 | 19.11 ± 16.33 | 17.91 ± 7.72 | 69.15 ± 8.70 | 27.05 ± 9.72 |
| 5129 | 140.64 ± 23.30 | 84.45 ± 18.84 | 119.85 ± 12.10 | 206.7 ± 29.44 | 94.65 ± 29.92 |
| Total | 65.47 ± 6.83 | 55.17 ± 7.32 | 78.87 ± 9.94 | 98.87 ± 7.52 | 60.15 ± 8.98 |

Supplementary Table 5. Number of good quality cotyledonary embryos available for germination before (0 mo) and after (6 mo) cold storage in Trial I.

| Line | clumps w/o prem. | | filters w/o prem. | | prematured clumps | | prematured filters | |
| --- | --- | --- | --- | --- | --- | --- | --- | --- |
|  | 0 mo | 6 mo | 0 mo | 6 mo | 0 mo | 6 mo | 0 mo | 6 mo |
| 1130 | 87 | 130 | 82 | 123 | 26 | 46 | 53 | 103 |
| 1548 | 121 | 161 | 159 | 177 | 45 | 52 | 99 | 99 |
| 2816 | 46 | 100 | 120 | 216 | 81 | 93 | 139 | 172 |
| 3492 | 87 | 133 | 232 | 238 | 242 | 270 | 94 | 118 |
| 5137 | 50 | 91 | 25 | 79 | 113 | 189 | 148 | 214 |
| Total | 391 | 615 | 618 | 833 | 507 | 650 | 533 | 706 |

Supplementary Table 6. Number of good quality cotyledonary embryos available for germination before (0 mo) and after (1 mo) cold storage in Trial II.

| Line | Selected Clumps | | Nonselected Clumps | | Nonselected clumps in prolif., ET selected for maturation | | Selected Filters | | Nonselected Filters | |
| --- | --- | --- | --- | --- | --- | --- | --- | --- | --- | --- |
|  | 0 mo | 1 mo | 0 mo | 1 mo | 0 mo | 1 mo | 0 mo | 1 mo | 0 mo | 1 mo |
| 290 | 8 | 14 | 5 | 7 | 3 | 4 | 18 | 26 | 16 | 14 |
| 623 | 79 | 103 | 77 | 98 | 98 | 116 | 71 | 81 | 55 | 71 |
| 655 | 120 | 135 | 71 | 79 | 125 | 123 | 189 | 184 | 96 | 106 |
| 1130 | 39 | 58 | 92 | 108 | 192 | 187 | 63 | 70 | 76 | 95 |
| 1375 | 127 | 145 | 90 | 96 | 88 | 122 | 172 | 174 | 83 | 88 |
| 1548 | 3 | 8 | 18 | 18 | 14 | 17 | 64 | 70 | 21 | 29 |
| 2816 | 17 | 23 | 37 | 58 | 29 | 41 | 80 | 100 | 8 | 15 |
| 2851 | 49 | 52 | 82 | 89 | 76 | 97 | 98 | 99 | 169 | 172 |
| 4262 | 44 | 70 | 26 | 55 | 46 | 108 | 47 | 89 | 9 | 23 |
| 4631 | 42 | 40 | 25 | 20 | 27 | 37 | 32 | 35 | 24 | 30 |
| 5111 | 51 | 59 | 18 | 19 | 17 | 28 | 66 | 81 | 26 | 29 |
| 5129 | 133 | 151 | 79 | 87 | 103 | 132 | 194 | 221 | 91 | 116 |
| Total | 712 | 858 | 620 | 734 | 818 | 1012 | 1094 | 1230 | 674 | 788 |

Supplementary Table 7. Survival of each line in all treatments 41 d after transplantation in Trial I. The number in parentheses represents the number of embryos transplanted

| Line | Clumps | Clumps, prem. | Filters | Filters, prem. |
| --- | --- | --- | --- | --- |
| 1130 | 90.7% (129) | 71.7% (46) | 82% (122) | 83.5% (103) |
| 1548 | 75.2% (133) | 58.5% (53) | 72.3% (177) | 71.7% (99) |
| 2816 | 89% (100) | 84.7% (98) | 95.3% (215) | 93% (172) |
| 3492 | 32.6% (132) | 68.1% (270) | 40.3% (236) | 73.8% (80) |
| 5137 | 60.5% (86) | 80.9% (188) | 76.6% (77) | 84.9% (258) |
| Total | 69.1% (580) | 73.7% (655) | 71% (827) | 83.6% (712) |

Supplementary Table 8. Survival of each line in all treatments 41 d after transplantation in Trial II. The number in parentheses represents the number of embryos transplanted

| Line | Selected Clumps | Nonselected Clumps | Clumps, random prolif., selected for maturation | Selected Filters | Nonselected Filters |
| --- | --- | --- | --- | --- | --- |
| 290 | 14.3% (14) | 11.1% (9) | 33.3% (12) | 19.2% (26) | 0% (2) |
| 623 | 33% (112) | 40.2% (97) | 38.8% (116) | 41.7% (72) | 32.4% (71) |
| 655 | 80.7% (135) | 64.6% (79) | 71.5% (123) | 80.8% (182) | 63.9% (108) |
| 1130 | 77.9% (131) | - (0) | 83.1% (178) | 79% (105) | 84.4% (96) |
| 1375 | 81.3% (144) | 83.3% (96) | 63.1% (122) | 70.7% (174) | 76.1% (88) |
| 1548 | 62.5% (8) | 88.9% (18) | 76.5% (17) | 80% (70) | 51.7% (29) |
| 2816 | 33.3% (27) | 40.3% (62) | 58.5% (42) | 40.9% (93) | 33.3% (15) |
| 2851 | 75% (52) | 52.2% (90) | 70.2% (178) | 63% (100) | 63.7% (91) |
| 4262 | 5.7% (70) | 19.3% (57) | 17.6% (108) | 6.7% (89) | 28.6% (21) |
| 4631 | 0% (40) | 0% (20) | 2.7% (37) | 0% (35) | 0% (30) |
| 5111 | 62.7% (59) | 77.8% (18) | 75% (28) | 61.9% (84) | 60.7% (28) |
| 5129 | 70.5% (149) | 59.1% (88) | 62.9% (132) | 55.7% (221) | 51.3% (117) |
| Total | 60.1% (941) | 58.0% (1251) | 53.0% (634) | 57.6% (696) | 58.0% (1093) |

**Supplementary Table 9 Percentage of factor effect and statistical significance (*: *p*<0.05, **: *p*<0.01; ***: *p*<0.001).**

| Compound | Storage effect | Genotype effect | Interaction storage:genotype effect |
| --- | --- | --- | --- |
| Stachyose | 80.0 *** | 5.4 *** | 10.9 *** |
| Raffinose | 82.2 *** | 3.9 *** | 9.1 *** |
| Sucrose | 71.9 *** | - | 11.3 ** |
| chiro-Inositol | 42.5 *** | 33.9 *** | 18.2 *** |
| myo-Inositol | 58.1 *** | - | 9.9 * |
| Glucose | 28.5 *** | 36.4 *** | 24.0 *** |
| Fructose | 31.3 *** | 30.4 *** | 24.3 *** |
| Galactose | 46.1 *** | 17.5 *** | 21.4 *** |
| Starch | 33.2 *** | 29.5 *** | 25.2 *** |
| tot. soluble carb. | 82.7 *** | 5.3 *** | 4.7 * |
| tot. carb. | 74.3 *** | 5.7 *** | 11.2 *** |
| Protein | 80.0 *** | 5.4 *** | 10.90 *** |

**Supplementary Table 10. The results are given as means ± SEM. Different letters indicate significant differences according to the multiple comparisons of means (*p* < 0.05, n = 5).**

| Compound | 0 | 4 | 8 | 26 | 61 | ZE |
| --- | --- | --- | --- | --- | --- | --- |
| Stachyose | 3.11 ± 0.8 a | 42.47 ± 12.9 ghj | 61.9 ± 20.5 lm | 26.38 ± 4.5 defg | 22.4 ± 7.8 bcf | 56.44 ± 14.5 jkm |
| Raffinose | 2.17 ± 0.8 a | 15.21 ± 4.6 abe | 24.16 ± 9.7 bdf | 30.01 ± 5.9 efh | 33.97 ± 7.7 fhi | 15.67 ± 9.4 acf |
| Sucrose | 38.93 ± 10.3 ghi | 43.56 ± 7.8 hj | 60.6 ± 13.7 km | 76.13 ± 8.3 n | 79.65 ± 11.9 n | 32.12 ± 9.2 defh |
| Glucose | 7.03 ± 3.4 ac | 7.11 ± 3.2 | 13.71 ± 8.1 acd | 7.05 ± 1.5 | 6.81 ± 1.6 ac | 2.05 ± 0.3 a |
| Galactose | 1.95 ± 0.6 | 2.68 ± 0.5 | 3.74 ± 0.8 | 2.63 ± 0.4 | nd | 2.87 ± 0.5 |
| Frutose | 7.44 ± 3.9 ac | 7.55 ± 3.5 ac | 15.98 ± 10.1 abe | 7.23 ± 2.3 ac | 6.76 ± 2.1 ac | 2.33 ± 0.1 ab |
| c-Inositol | 2.41 ± 0.6 | 1.96 ± 0.3 | 2.65 ± 0.4 | 2.69 ± 0.7 | nd | 1.58 ± 0.2 |
| m-Inositol | 4.28 ± 0.8 | 2.33 ± 0.3 | 3.33 ± 0.6 | 4.08 ± 0.9 | 2.65 ± 0.3 | 2.06 ± 0.3 |
| Starch | 55.36 ± 17.4 jkl | 51.36 ± 13.2 ijkl | 45.31 ± 11.7 hk | 71.59 ± 13.5 mn | 79.95 ± 29.2 n | 6.24 ± 5.2 ab |
| tot sol. carb. | 66.44 ± 13.9 | 121.5 ± 23.8 | 184.1 ± 36.6 | 154.88 ± 14 | 152.2 ± 11.7 | 113.9 ± 30 |
| tot carb. | 121.8 ± 26.5 | 172.8 ± 28.1 | 229.4 ± 43.7 | 226.47 ± 23.6 | 232.2 ± 24.2 | 120.1 ± 27.5 |
| Protein | 95 ± 22.7 AB | 116.2 ± 27.2 BC | 119 ± 13.7 BC | 130.3 ± 18.5 C | 74.2 ± 9.7 A | 118.8 ± 21.5 BC |
